# Clinicopathological and Prognostic Significance of TROP2 and HER3 Expression in Early-Stage Cervical Carcinoma

**DOI:** 10.3390/diagnostics16172871

**Published:** 2026-09-07

**Authors:** Dilay Karademir, Nazan Yurtcu, Ramazan Oguz Yüceer

**Affiliations:** 1 Department of Obstetrics and Gynecology, Sivas Cumhuriyet University School of Medicine, Sivas 58140, Turkey; nyurtcu58@gmail.com; 2 Department of Pathology, Sivas Cumhuriyet University School of Medicine, Sivas 58140, Turkey; r.yuceer66@hotmail.com

**Keywords:** cervical carcinoma, TROP2, HER3, immunohistochemistry, prognostic biomarker, targeted therapy, prognosis

## Abstract

**Background:** Our objective was to evaluate the prognostic value of TROP2 and HER3 expression in patients with early-stage cervical carcinoma undergoing primary surgical treatment. **Methods:** We retrospectively analyzed 54 patients with FIGO 2018 stage IA–IIA cervical carcinoma who underwent radical surgery between January 2016 and December 2025. TROP2 and HER3 expression were assessed using immunohistochemistry and a standardized immunoreactivity scoring system. Associations with clinicopathological characteristics were examined, and survival outcomes were analyzed using Kaplan–Meier estimates and Cox proportional hazards models. **Results:** High TROP2 and HER3 expression was identified in 63.0% and 37.0% of tumors, respectively. Neither biomarker was associated with the conventional clinicopathological variables. After a median follow-up of 88.2 months, 22 patients (40.7%) developed recurrent disease and 18 (33.3%) died. Patients with high TROP2 expression had significantly shorter overall and disease-free survival than those with low expression. After adjustment for established prognostic factors, high TROP2 expression remained independently associated with disease recurrence (HR 5.00, 95% CI 1.28–19.57; *p* = 0.021). In contrast, HER3 expression was not independently associated with overall or disease-free survival rates. Older age and advanced FIGO stage were independently associated with poorer outcomes. **Conclusions:** TROP2 expression identifies a subgroup of patients with early-stage cervical carcinoma who are at an increased risk of recurrence despite primary surgical treatment. Unlike HER3, TROP2 provided prognostic information beyond conventional clinicopathological factors, supporting its potential role in postoperative risk stratification. These findings also reinforce the rationale for further investigation of TROP2 as a therapeutic target in cervical carcinoma.

## 1. Introduction

Cervical carcinoma remains one of the most common malignancies and is a leading cause of cancer-related death among women worldwide [1]. Although the widespread implementation of human papillomavirus (HPV) vaccination and cervical screening programs has substantially reduced disease incidence in many high-income countries, cervical cancer continues to impose a considerable health burden in low- and middle-income regions, where access to preventive and early diagnostic services remains limited [2]. Persistent infection with high-risk HPV types, particularly HPV16 and HPV18, is the primary driver of cervical cancer. The viral oncoproteins E6 and E7 promote malignant transformation by inactivating the tumor suppressor proteins p53 and retinoblastoma (Rb), leading to genomic instability, dysregulated cell cycle control, and uncontrolled cellular proliferation [3].

Most patients with early-stage cervical carcinoma achieve favorable outcomes after radical surgery, with or without adjuvant treatment. Nevertheless, disease recurrence remains the leading cause of treatment failure and cancer-related mortality. Despite apparently curative treatment, some patients experience locoregional or distant relapse, highlighting the considerable biological heterogeneity of this disease. At present, postoperative risk stratification is primarily based on established clinicopathological factors, including FIGO stage, tumor size, histological subtype, depth of stromal invasion, lymphovascular space invasion, lymph node involvement, and surgical margin status [4]. Although these parameters are essential for guiding adjuvant treatment and estimating prognosis, they do not fully explain the variability in clinical outcomes observed among patients with similar pathological characteristics. Consequently, there is growing interest in identifying reliable molecular biomarkers that can complement conventional prognostic factors, improve risk stratification, and support more individualized management of patients with early-stage cervical carcinoma [5].

The growing emphasis on precision oncology has increased interest in biomarkers that can improve prognostic assessment and serve as potential therapeutic targets. One such biomarker is the trophoblast cell surface antigen 2 (TROP2), a transmembrane glycoprotein encoded by the *TACSTD2* gene. TROP2 plays an important role in tumor progression by promoting cell proliferation, migration, invasion, and epithelial–mesenchymal transition through the activation of several oncogenic signaling pathways, including PI3K/AKT, MAPK/ERK, and NF-κB [6]. TROP2 overexpression has been reported in a wide range of epithelial malignancies and has consistently been associated with aggressive tumor behavior, increased metastatic potential, and poor clinical outcomes [7,8,9]. More recently, the clinical success of TROP2-targeted antibody–drug conjugates has further highlighted its therapeutic relevance, making TROP2 an attractive biomarker for both prognostic evaluation and targeted treatment strategies in cervical carcinoma [10,11].

Another biomarker of growing interest is the human epidermal growth factor receptor 3 (HER3, *ERBB3*), a member of the ERBB receptor family. Although HER3 has minimal intrinsic kinase activity, it exerts biological effects by forming heterodimers with other ERBB receptors, particularly HER2, leading to the activation of the PI3K/AKT signaling pathway. Through these interactions, HER3 contributes to tumor cell proliferation, survival, invasion, and resistance to anticancer therapy [12]. Increased HER3 expression has been linked to unfavorable clinicopathological features and poor prognosis in several solid malignancies [13,14,15,16]. However, its prognostic significance in cervical carcinoma remains uncertain, as published studies have reported conflicting results [17].

Given their distinct but complementary roles in tumor progression, TROP2 and HER3 may provide prognostic information beyond that offered by conventional clinicopathological factors. A better understanding of their clinical significance could improve risk stratification and help identify patients at an increased risk of recurrence after primary surgery. Therefore, the present study evaluated the immunohistochemical expression of TROP2 and HER3 in a cohort of patients with early-stage cervical carcinoma and investigated their associations with clinicopathological characteristics, disease recurrence, progression-free survival, and overall survival. We also sought to determine whether these biomarkers provide independent prognostic information that could support more individualized postoperative management and guide future biomarker-driven therapeutic strategies in patients.

## 2. Materials and Methods

### 2.1. Study Design and Patient Selection

This retrospective cohort study was conducted at the Departments of Pathology and Obstetrics and Gynecology of Sivas Cumhuriyet University Faculty of Medicine. The study included patients who underwent primary surgical treatment for cervical carcinoma between January 2016 and December 2025.

A total of 75 consecutive patients with histopathologically confirmed cervical carcinoma were initially identified through the institutional electronic pathology archive and hospital information system. Clinical records, operative reports, pathology reports, radiological examinations, treatment records, and follow-up data were comprehensively reviewed.

Patients were eligible if they fulfilled all of the following criteria:

- Histologically confirmed primary cervical carcinoma;
- FIGO 2018 stage IA–IIA disease;
- Primary treatment with radical hysterectomy and pelvic lymph node dissection, with or without para-aortic lymphadenectomy;
- Availability of adequate formalin-fixed paraffin-embedded (FFPE) tissue for immunohistochemical analysis;
- Complete clinicopathological and follow-up data.

Patients were excluded if they had received neoadjuvant chemotherapy, radiotherapy, or concurrent chemoradiotherapy before surgery (*n* = 2); had insufficient tumor tissue available for immunohistochemical staining (*n* = 6); had incomplete clinicopathological or follow-up information (*n* = 10); or had synchronous malignancies or distant metastatic disease at diagnosis (*n* = 3).

**Figure 1:**
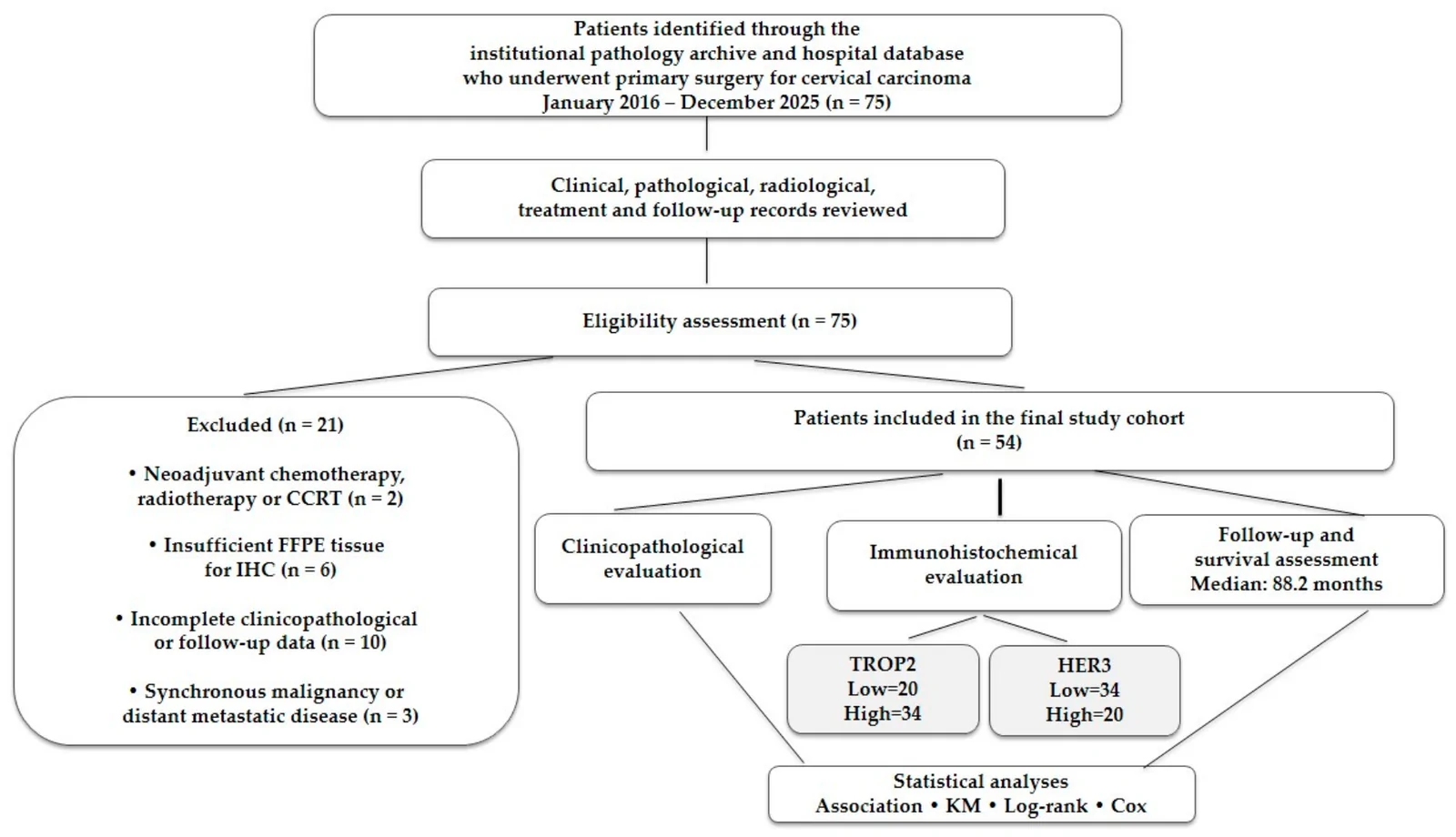
Study flow diagram illustrating patient selection and study design.

After applying these eligibility criteria, 54 patients were included in the final study cohort and underwent clinicopathological, immunohistochemical, and survival analyses. All patient data were anonymized before statistical analysis to ensure confidentiality (Figure 1).

### 2.2. Follow-Up and Outcome Assessment

All patients were managed according to the contemporary National Comprehensive Cancer Network (NCCN) guidelines and institutional protocols for early-stage cervical carcinoma [18]. Primary treatment consisted of radical hysterectomy with pelvic lymph node dissection, with or without para-aortic lymphadenectomy. Postoperative adjuvant radiotherapy, concurrent platinum-based chemoradiotherapy, or observation was administered according to pathological risk factors and multidisciplinary tumor board recommendations.

The primary study endpoints were disease-free survival (DFS) and overall (OS) survival. DFS was defined as the interval from primary surgery to the first documented recurrence, disease progression, death from any cause, or last follow-up. OS was defined as the interval from primary surgery to death from any cause or the last follow-up. Mortality was evaluated based on death from any cause rather than cancer-specific death. Patients without recurrence who were alive at the last follow-up were censored on the date of their most recent clinical assessment. All survival analyses were based exclusively on data derived from the study cohort, without the incorporation of any publicly available or external datasets.

### 2.3. Histopathological Evaluation

All surgical specimens were immediately fixed in 10% neutral-buffered formalin for 24–48 h after surgical resection and subsequently processed according to standardized pathology protocols. Tissue samples were processed using an automated tissue processor, embedded in paraffin, sectioned to 4-μm thickness, and stained with hematoxylin and eosin (H&E).

Histopathological diagnosis was confirmed according to the 2020 World Health Organization (WHO) Classification of Female Genital Tumors [19], whereas pathological staging was reassessed according to the 2018 International Federation of Gynecology and Obstetrics (FIGO) staging system [20].

The histological subtypes were classified as squamous cell carcinoma or adenocarcinoma. Tumor grade was recorded according to the original pathological diagnosis and re-evaluated when required.

### 2.4. Immunohistochemical Evaluation of TROP2 and HER3 Expression

Representative formalin-fixed, paraffin-embedded (FFPE) tissue blocks were selected after a comprehensive review of all available hematoxylin and eosin (H&E)-stained slides. For each case, a tissue block containing the largest area of viable invasive carcinoma with optimal fixation and preservation was selected for immunohistochemical analysis. Areas predominantly composed of carcinoma in situ, necrosis, hemorrhage, fibrosis, inflammatory infiltrates, cautery artifacts, or tissue folds were excluded. In tumors showing intratumoral heterogeneity, multiple blocks were reviewed, and the block most representative of the invasive tumor component was selected for the analysis.

Immunohistochemical staining was performed on 4-µm-thick sections using a fully automated immunostaining platform (Ventana Benchmark Ultra; Roche Diagnostics, Basel, Switzerland). The sections were incubated with primary antibodies against TROP2 (mouse monoclonal antibody, Clone B-9, 1:100 dilution, Santa Cruz Biotechnology, Santa Cruz, CA, USA) and HER3 (rabbit monoclonal antibody, clone D22C5, 1:100 dilution; Cell Signaling Technology, Danvers, MA, USA). Appropriate positive controls were included in each staining run, consisting of normal breast epithelium for TROP2 and breast carcinoma tissue for HER3, according to the manufacturers’ recommendations. Negative controls were prepared by omitting the primary antibodies.

All stained slides were independently evaluated by experienced gynecologic pathologists (R.O.Y.) who were blinded to the clinicopathological data and the patient outcomes. For TROP2, membranous and/or cytoplasmic staining of invasive tumor cells was assessed using a semi-quantitative method. Staining intensity was graded as 0 (absent), 1 (weak), 2 (moderate), or 3 (strong), while the proportion of positive tumor cells was scored as 0 (0%), 1 (1–19%), 2 (20–50%), and 3 (>50%). The immunoreactivity score (IRS) was calculated by multiplying the intensity and proportion scores, yielding a total score ranging from 0 to 9 [21]. Tumors with an IRS < 6 were classified as having low TROP2 expression, whereas those with an IRS ≥ 6 were classified as having high TROP2 expression.

For HER3, only membranous staining was considered positive. Staining intensity was scored as 0 (absent), 1 (weak), 2 (moderate), or 3 (strong), and the proportion of positive tumor cells was graded as 0 (0%), 1 (1–10%), 2 (11–30%), 3 (31–50%), and 4 (>50%). The immunoreactivity score (IRS) was calculated by multiplying the intensity and proportion scores [22]. Tumors with an IRS < 6 were classified as having low HER3 expression, whereas those with an IRS ≥ 6 were considered to have high HER3 expression.

Although the proportion scores differed between TROP2 and HER3, the scoring systems were based on previously published biomarker-specific methods rather than on the distribution of expression in our cohort [21,22]. These differences reflect the established immunohistochemical assessment criteria for each marker. However, an IRS of ≥6 was used to define high expression for both biomarkers. The scoring criteria and cutoffs were therefore defined a priori and were not selected or modified according to the survival outcomes or biomarker distribution in the present cohort.

### 2.5. Statistical Analysis

Statistical analyses were performed using IBM SPSS Statistics version 27.0 (IBM Corp., Armonk, NY, USA) and R software version 4.5.2 (R Foundation for Statistical Computing, Vienna, Austria). The normality of continuous variables was assessed using the Shapiro–Wilk test. Normally distributed variables are presented as mean ± standard deviation (SD), whereas non-normally distributed variables are expressed as median (interquartile range [IQR]). Categorical variables were summarized as frequencies and percentages.

Comparisons between biomarker expression groups were performed using the independent-samples Student’s *t*-test or Mann–Whitney U test, as appropriate. Categorical variables were compared using Pearson’s chi-square or Fisher’s exact tests.

Disease-free survival (DFS) and overall survival (OS) were estimated using the Kaplan–Meier method and compared using the log-rank test. Prognostic factors associated with DFS and OS were initially evaluated using univariate Cox proportional hazards regression analysis. Variables with *p* < 0.10 in the univariable analysis, together with clinically relevant variables, were included in the multivariable Cox regression models.

For each survival endpoint, two multivariate models were constructed separately for TROP2 and HER3. Model 1 included age, menopausal status, FIGO stage, histological subtype, tumor size, lymphovascular space invasion (LVSI), and adjuvant treatment status. Model 2 included all variables in Model 1, together with either TROP2 or HER3 expression, to determine the independent prognostic contribution of each biomarker.

Hazard ratios (HRs) and 95% confidence intervals (95% CIs) were calculated for all Cox models. The proportional hazards assumption was evaluated using scaled Schoenfeld residuals for each covariate as well as for the overall model. A *p* value > 0.05 was considered to indicate no significant departure from proportionality. Both individual covariate and global tests were examined, and no significant violations of the proportional hazards assumption were identified. Statistical significance was set at *p* < 0.05.

## 3. Results

This study included 54 patients with early-stage cervical carcinoma. The mean age was 57.5 ± 12.2 years, and the median gravida and parity were 3 (IQR, 2–4). Most patients were postmenopausal (72.2%), and 51.9% had at least one comorbidity. HPV18 (44.4%) and HPV16 (33.3%) were the predominant HPV genotypes. Squamous cell carcinoma accounted for 81.5% of cases, and 38.9% demonstrated lymphovascular space invasion. Primary treatment consisted of radical hysterectomy. Eight patients underwent surgery without pelvic lymph node dissection and had no evidence of nodal involvement at initial evaluation. Para-aortic lymph node involvement was present in 12 patients and received adjuvant treatment (68.5% of patients). Chemotherapy was administered to 94.4% of patients (Table 1).

**Table 1:** Baseline clinicopathological characteristics of patients with early-stage cervical carcinoma.

Characteristic	Overall (*n* = 54)
Age, years	57.5 ± 12.2
Gravida	3 (2–4)
Parity	3 (2–4)
Menopausal status	
Premenopausal	15 (27.8)
Postmenopausal	39 (72.2)
Comorbidity	
Absent	26 (48.1)
Present	28 (51.9)
HPV status	
Negative	10 (18.5)
HPV16	18 (33.3)
HPV18	24 (44.4)
Other	2 (3.7)
FIGO 2018 stage	
IA1	10 (18.5)
IA2	15 (27.8)
IB1	7 (13.0)
IB2	4 (7.4)
IB3	10 (18.5)
IIA1	4 (7.4)
IIA2	4 (7.4)
Tumor size	
<2 cm	24 (44.4)
2–4 cm	12 (22.2)
>4 cm	18 (33.3)
Histological subtype	
Squamous cell carcinoma	44 (81.5)
Adenocarcinoma	10 (18.5)
LVSI	
Absent	33 (61.1)
Present	21 (38.9)
Surgical procedure	
Radical hysterectomy	8 (14.8)
Radical hysterectomy + pelvic lymph node dissection	46 (85.2)
Adjuvant treatment	
No	17 (31.5)
Yes	37 (68.5)
Chemotherapy	
No	3 (5.6)
Yes	51 (94.4)

**Figure 2:**
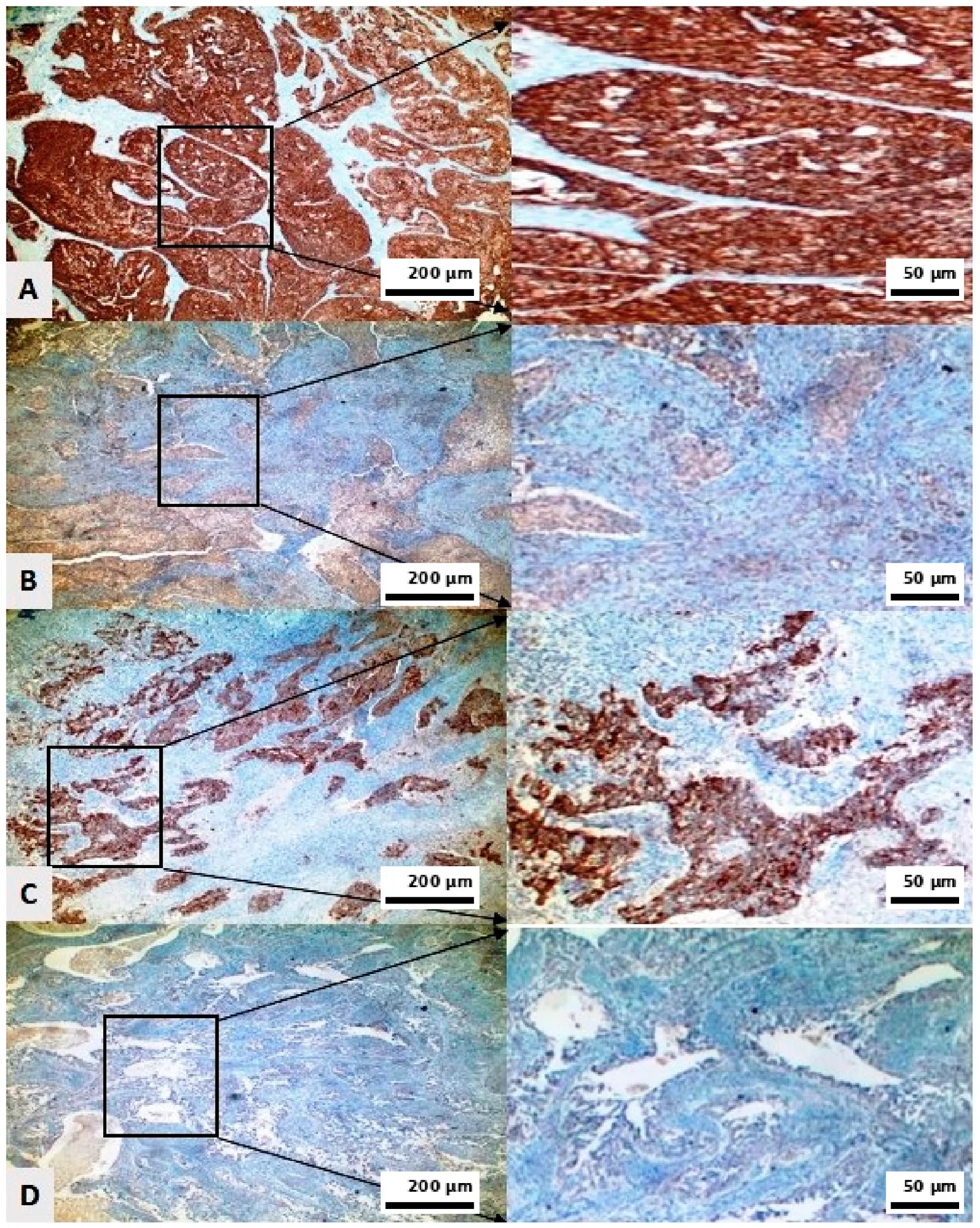
Representative immunohistochemical staining patterns of TROP2 and HER3 in cervical carcinoma. (**A**) High TROP2 expression with strong membranous and cytoplasmic staining in tumor cells. (**B**) Negative/low TROP2 expression with absent or minimal staining. (**C**) High HER3 expression with prominent membranous staining in tumor cells. (**D**) Negative/low HER3 expression with absent or weak membranous staining. For each case, the left panel shows the low-magnification image (scale bar, 200 µm), with the boxed area shown at higher magnification in the corresponding right panel (scale bar, 50 µm).

Among the 54 patients included in the study, low TROP2 expression was observed in 20 (37.0%), whereas high TROP2 expression was detected in 34 (63.0%). In contrast, HER3 expression was classified as low in 34 patients (63.0%) and high in 20 patients (37.0%) (Figure 2).

The associations between TROP2 and HER3 expression and clinicopathological characteristics are shown in Table 2. Patients with low and high TROP2 expression levels showed comparable demographic and clinicopathological characteristics. The mean age was similar between the TROP2-low and TROP2-high groups (57.2 ± 10.2 vs. 57.7 ± 13.4 years, *p* = 0.897). No significant differences were observed in gravida, parity, menopausal status, comorbidity, HPV genotype, FIGO 2018 stage, tumor size, histological subtype, or lymphovascular space invasion (*p* > 0.05). Similarly, the distribution of surgical procedures and adjuvant treatments, including radiotherapy and chemotherapy, did not differ significantly between the groups.

Similarly, HER3 expression was not significantly associated with any baseline clinicopathological characteristics. Patients with high and low HER3 expression had comparable age, reproductive history, menopausal status, comorbidity status, HPV subtype distribution, FIGO stage, tumor size, histological subtype, LVSI status, and surgical management (*p* > 0.05). Although patients with low HER3 expression more frequently received adjuvant radiotherapy (76.5% vs. 55.0%, *p* = 0.091), these differences did not reach statistical significance. Similarly, adjuvant chemotherapy was administered to nearly all patients in both HER3 expression groups (97.1% vs. 90.0%, *p* = 0.306).

During a median follow-up of 88.2 months, 18 (33.3%) patients died and 36 (66.7%) remained alive. Disease recurrence occurred in 22 patients (40.7%), whereas 32 patients (59.3%) remained recurrence free. The median overall survival (OS) for the entire cohort was 89.1 months (95% CI: 75.2–103.1), and the median disease-free survival (DFS) was 80.5 months (95% CI: 66.0–95.2).

**Figure 3:**
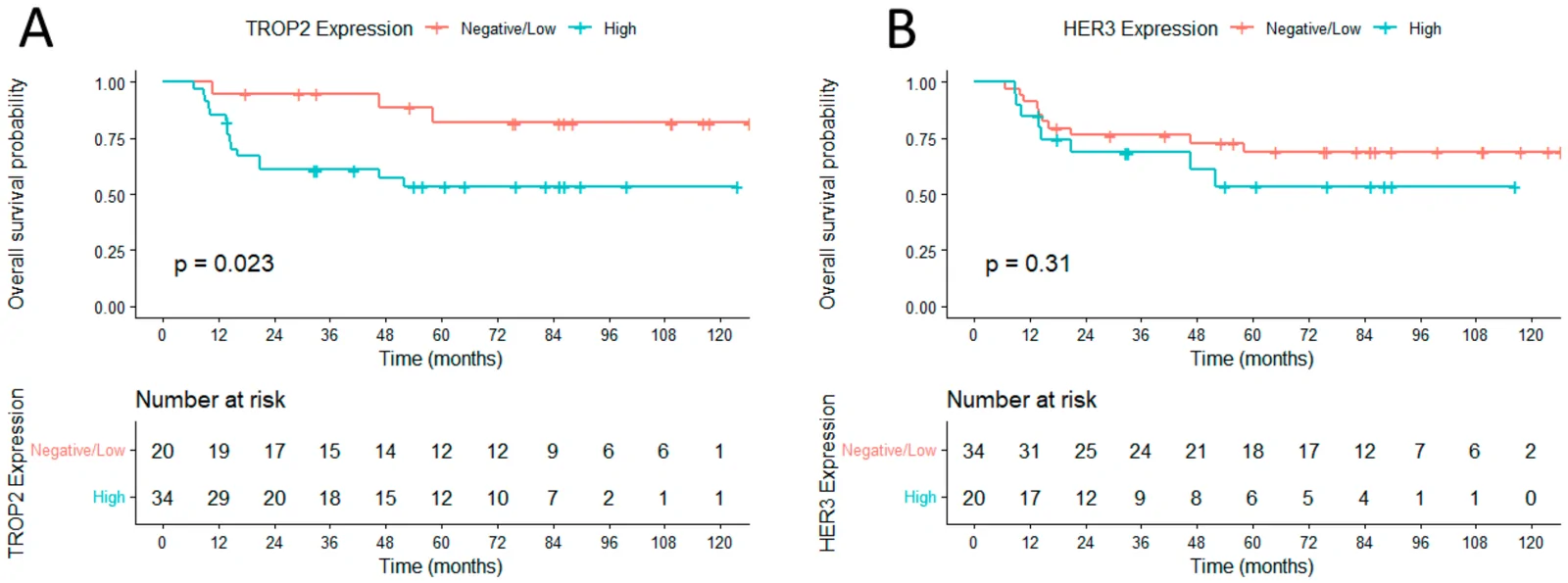
Kaplan–Meier curves for overall survival according to TROP2 and HER3 expression. (**A**) Overall survival according to TROP2 expression (negative/low, *n* = 20; high, *n* = 34). (**B**) Overall survival according to HER3 expression (negative/low, *n* = 34; high, *n* = 20). Numbers below each curve indicate the number of patients at risk at the corresponding time points. Differences between groups were assessed using the log-rank test.

Kaplan–Meier survival analysis demonstrated that patients with negative/low TROP2 expression had a significantly longer median OS than those with high TROP2 expression (110.9 months [95% CI: 94.8–127.0] vs. 75.1 months [95% CI: 56.7–93.4], respectively; log-rank *p* = 0.023), indicating that high TROP2 expression was associated with a poorer overall survival. Regarding HER3 expression, the median OS was 94.3 months (95% CI: 77.5–111.1) in the negative/low HER3 expression group and 73.9 months (95% CI: 51.5–96.2) in the high HER3 expression group. However, this difference was not statistically significant (log-rank *p* = 0.310) (Figure 3).

**Figure 4:**
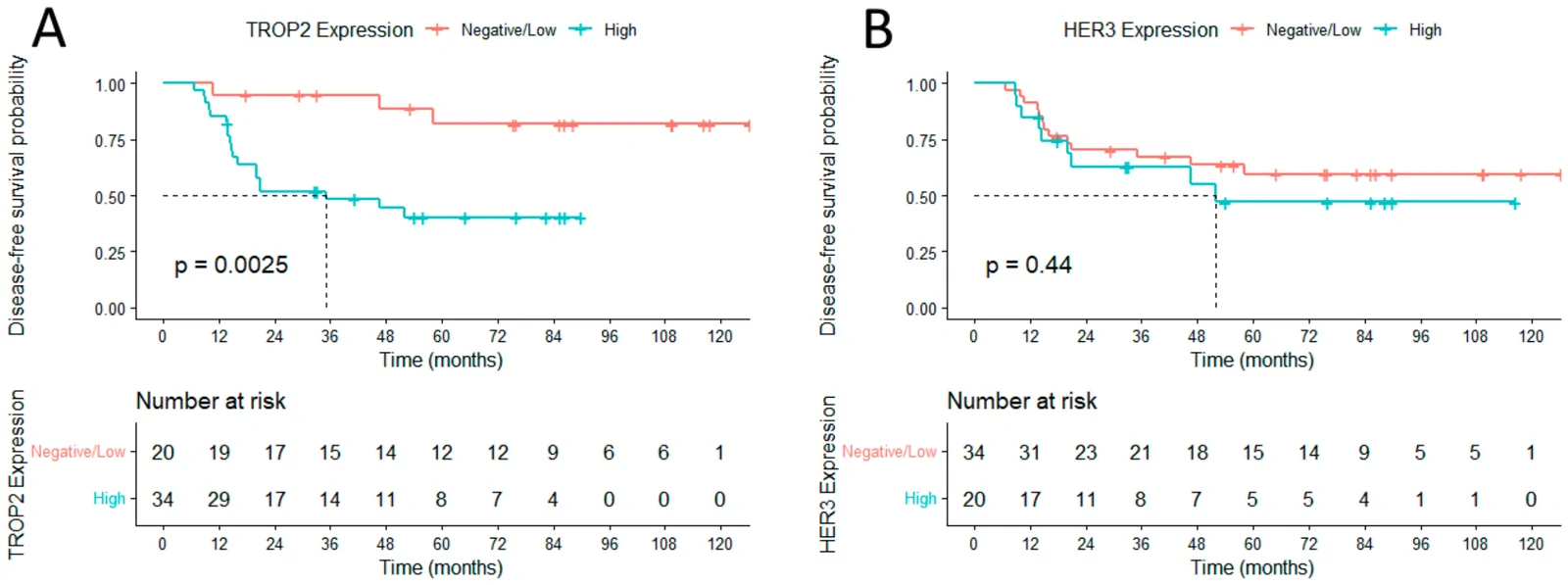
Kaplan–Meier curves for disease-free survival according to TROP2 and HER3 expression. (**A**) Disease-free survival according to TROP2 expression (negative/low, *n* = 20; high, *n* = 34). (**B**) Disease-free survival according to HER3 expression (negative/low, *n* = 34; high, *n* = 20). Numbers below each curve indicate the number of patients at risk at the corresponding time points. Differences between groups were assessed using the log-rank test.

Kaplan–Meier analysis further demonstrated that patients with negative/low TROP2 expression had a significantly longer median DFS than those with high TROP2 expression (110.9 months [95% CI: 94.8–127.0] vs. 48.4 months [95% CI: 36.0–60.7], respectively; log-rank *p* = 0.003), indicating that elevated TROP2 expression was associated with an increased risk of disease recurrence. In a separate analysis of HER3 expression, the median DFS was 84.7 months (95% CI: 66.9–102.5) in the negative/low HER3 expression group and 68.0 months (95% CI: 45.4–90.7) in the high HER3 expression group. However, the difference between the two groups was not statistically significant (log-rank *p* = 0.440) (Figure 4).

Univariable Cox proportional hazards regression analyses were performed to identify clinicopathological factors associated with overall survival (OS) and disease-free survival (DFS). For OS, high TROP2 expression was significantly associated with an increased risk of mortality (HR = 2.01, 95% CI: 1.12–3.58, *p* = 0.019). HER3 expression demonstrated a trend toward worse OS but did not reach statistical significance (HR = 1.69, 95% CI: 0.96–2.98, *p* = 0.071). FIGO stage also showed a borderline association with OS (HR = 1.17, 95% CI: 1.00–1.36, *p* = 0.051). No significant associations were observed between OS and age, gravida, parity, menopausal status, comorbidity, HPV status, tumor size, histological subtype, lymphovascular invasion, surgical approach, adjuvant chemotherapy, and adjuvant radiotherapy (*p* > 0.05). For DFS, elevated TROP2 expression was identified as a significant predictor of disease recurrence (HR = 2.97, 95% CI: 1.56–5.64, *p* < 0.001). Although the surgical approach showed a borderline association with DFS (HR = 2.28, 95% CI: 0.94–5.56, *p* = 0.069), no statistically significant relationships were observed for HER3 expression or the remaining clinicopathological variables (*p* > 0.05) (Table 3).

Multivariable Cox proportional hazards regression analyses were performed using three sequential models to evaluate the independent prognostic value of TROP2 and HER3 expression for overall survival (OS). In Model 1, which included clinicopathological variables only, older age (HR = 1.07, 95% CI: 1.02–1.13, *p* = 0.011), advanced FIGO stage (HR = 1.80, 95% CI: 1.33–2.43, *p* < 0.001), and lymphovascular invasion (HR = 1.06, 95% CI: 0.01–1.31, *p* < 0.001) were independently associated with OS. After the addition of TROP2 expression in Model 2, age and FIGO stage remained significant independent predictors, whereas high TROP2 expression was not independently associated with OS (HR = 2.40, 95% CI: 0.61–9.47, *p* = 0.213). In Model 3, which additionally incorporated HER3 expression, the associations between age, FIGO stage, and lymphovascular invasion remained unchanged. Neither TROP2 (HR = 2.42, 95% CI: 0.59–10.00, *p* = 0.221) nor HER3 expression (HR = 0.96, 95% CI: 0.27–3.39, *p* = 0.950) emerged as independent prognostic factors for OS after adjustment for clinicopathological variables (Table 4).

Multivariable Cox proportional hazards regression analyses were performed using three sequential models to determine the independent prognostic significance of TROP2 and HER3 expression for disease-free survival (DFS). In Model 1, older age (HR = 1.06, 95% CI: 1.01–1.11, *p* = 0.026), advanced FIGO stage (HR = 1.56, 95% CI: 1.18–2.06, *p* = 0.002), and lymphovascular invasion (HR = 1.09, 95% CI: 0.02–1.35, *p* < 0.001) were identified as independent predictors of DFS. Following the inclusion of TROP2 expression in Model 2, age and FIGO stage remained independently associated with DFS, while high TROP2 expression emerged as an independent adverse prognostic factor, conferring a 4.66-fold increased risk of disease recurrence (HR = 4.66, 95% CI: 1.26–17.17, *p* = 0.021). In Model 3, after additional adjustment for HER3 expression, the prognostic significance of TROP2 remained unchanged (HR = 5.00, 95% CI: 1.28–19.57, *p* = 0.021), whereas HER3 expression was not independently associated with DFS (HR = 0.80, 95% CI: 0.25–2.52, *p* = 0.701). These findings indicate that TROP2, but not HER3, provides independent prognostic information for disease recurrence beyond established clinicopathological variables (Table 5).

## 4. Discussion

In this study, we examined the clinicopathological and prognostic significance of TROP2 and HER3 expression in early-stage cervical carcinoma. Neither marker was significantly associated with the clinicopathological characteristics evaluated. High TROP2 expression, however, was associated with poorer overall and disease-free survival and with an increased risk of recurrence after adjustment for clinicopathological factors. HER3 expression showed no independent association with survival outcomes. These findings point to a potentially greater prognostic relevance of TROP2 in early-stage cervical carcinoma.

The relationship between TROP2 expression and recurrence, in the absence of clear associations with conventional pathological features, may reflect differences in the underlying biology of these tumors. Experimental studies have linked TROP2 to tumor cell proliferation, invasion, migration, and epithelial–mesenchymal transition through signaling pathways such as PI3K/AKT and MAPK/ERK [23,24]. These biological effects may partly account for the poorer disease-free survival observed in patients with high TROP2 expression. TROP2 may therefore capture aspects of tumor behavior that are not fully represented by routine clinicopathological parameters.

Our findings are broadly consistent with those of Hiller et al., who reported an association between TROP2 overexpression and unfavorable survival in cervical cancer [25]. In contrast to their study, we did not observe a significant relationship between TROP2 expression and clinicopathological features. Differences in patient characteristics and methodological factors, including immunohistochemical assessment and scoring criteria, may explain these differences [26]. Despite these variations, the association between TROP2 expression and recurrence in our cohort warrants further investigation of its potential role in postoperative risk assessment.

The findings for HER3 were less conclusive. Although high HER3 expression showed a tendency toward poorer overall survival, it was not independently associated with clinical outcomes. This differs from the findings of Chang et al., who identified HER3 overexpression as an independent predictor of both disease-free and overall survival in cervical cancer [27]. Differences in patient populations, treatment approaches, antibodies, scoring methods, and cut-off definitions may contribute to these conflicting results. The prognostic significance of HER3 in cervical cancer therefore remains uncertain and requires confirmation in independent cohorts.

TROP2 has also received increasing attention as a therapeutic target, particularly with the development of TROP2-directed antibody–drug conjugates, some of which have shown antitumor activity in recurrent or metastatic cervical cancer [28]. Our study, however, focused on the prognostic significance of TROP2 and did not evaluate treatment response. The present findings therefore cannot establish a predictive role for TROP2 or demonstrate benefit from TROP2-directed therapy.

This study had several limitations. The retrospective single-center design may have introduced a selection bias and limited the generalizability of the findings. The relatively small sample size, particularly for subgroup analyses, may have reduced the statistical power to detect modest associations, especially for HER3. The limited number of recurrence and death events also increased the potential for overfitting in the multivariable Cox models and may have affected the stability of the hazard ratio estimates. Therefore, these results, particularly those with wide confidence intervals, should be interpreted cautiously. Furthermore, our cohort was limited to surgically treated patients with early-stage cervical carcinoma; therefore, the findings may not be applicable to patients with more advanced disease. Finally, the lack of external validation and functional molecular analyses precluded confirmation of the biological mechanisms underlying the prognostic role of TROP2 and HER3. Future multicenter prospective studies with larger cohorts are required to validate these findings.

## 5. Conclusions

In summary, our findings indicate that TROP2 may have prognostic value in early-stage cervical carcinoma. High TROP2 expression was independently associated with an increased risk of recurrence, whereas HER3 expression showed no significant association with clinical outcomes. These findings suggest that TROP2 may help identify patients at higher risk of recurrence. However, treatment response was not assessed in this study, and our results therefore do not establish a predictive or therapeutic role for TROP2. Prospective studies with larger cohorts and treatment-response data are needed to confirm its prognostic value and explore its potential relevance as a therapeutic biomarker.

## Figures and Tables

**Table 2:** Association of TROP2 and HER3 expression with clinicopathological characteristics.

Variable	TROP2 Low (*n* = 20)	TROP2 High (*n* = 34)	*p*	HER3 Low (*n* = 34)	HER3 High (*n* = 20)	*p*
Age, years	57.2 ± 10.2	57.7 ± 13.4	0.897	58.2 ± 11.3	56.3 ± 13.9	0.577
Gravida	3 (2–4)	3 (2–5)	0.331	3.5 (2–5)	3 (2–4)	0.298
Parity	3 (2–3)	3 (2–5)	0.176	3 (2–4)	3 (2–3)	0.214
Menopausal status						
Premenopausal	5 (25.0)	10 (29.4)	0.491	8 (23.5)	7 (35.0)	0.274
Postmenopausal	15 (75.0)	24 (70.6)		26 (76.5)	13 (65.0)	
Comorbidity						
Absent	12 (60.0)	14 (41.2)	0.146	15 (44.1)	11 (55.0)	0.312
Present	8 (40.0)	20 (58.8)		19 (55.9)	9 (45.0)	
HPV status						
Negative	4 (20.0)	6 (17.7)		5 (14.7)	5 (25.0)	
HPV16	8 (40.0)	10 (29.4)	0.569	12 (35.3)	6 (30.0)	0.569
HPV18	7 (35.0)	17 (50.0)		16 (47.1)	8 (40.0)	
Other	1 (5.0)	1 (2.9)		1 (2.9)	1 (5.0)	
FIGO 2018 stage						
IA1	5 (25.0)	5 (14.6)		7 (20.7)	3 (15.0)	
IA2	5 (25.0)	10 (29.4)		10 (29.4)	5 (25.0)	
IB1	3 (15.0)	4 (11.8)		5 (14.7)	2 (10.0)	
IB2	2 (10.0)	2 (5.9)	0.171	3 (8.8)	1 (5.0)	0.153
IB3	5 (25.0)	5 (14.7)		6 (17.6)	4 (20.0)	
IIA1	0	4 (11.8)		2 (5.9)	2 (10.0)	
IIA2	0	4 (11.8)		1 (2.9)	3 (15.0)	
Tumor size						
<2 cm	10 (50.0)	14 (41.2)		16 (47.0)	8 (40.0)	
2–4 cm	4 (20.0)	8 (23.5)	0.571	7 (20.6)	5 (25.0)	0.697
>4 cm	6 (30.0)	12 (35.3)		11 (32.4)	7 (35.0)	
Histological subtype						
Squamous cell carcinoma	15 (75.0)	29 (85.3)	0.278	28 (82.4)	16 (80.0)	0.550
Adenocarcinoma	5 (25.0)	5 (14.7)		6 (17.6)	4 (20.0)	
LVSI						
Absent	11 (55.0)	22 (64.7)	0.337	20 (58.8)	13 (65.0)	0.439
Present	9 (45.0)	12 (35.3)		14 (41.2)	7 (35.0)	
Surgical procedure						
Radical hysterectomy	5 (25.0)	3 (8.8)	0.113	6 (17.6)	2 (10.0)	0.366
Radical hysterectomy + pelvic lymph node dissection	15 (75.0)	31 (91.2)		28 (82.4)	18 (90.0)	
Adjuvant radiotherapy						
No	7 (35.0)	10 (29.4)	0.447	8 (23.5)	9 (45.0)	0.091
Yes	13 (65.0)	24 (70.6)		26 (76.5)	11 (55.0)	
Adjuvant chemotherapy						
No	1 (5.0)	2 (5.9)	0.694	1 (2.9)	2 (10.0)	0.306
Yes	19 (95.0)	32 (94.1)		33 (97.1)	18 (90.0)	

**Table 3:** Univariable Cox proportional hazards regression analyses for overall survival and disease-free survival.

Variable	Overall Survival HR (95% CI)	*p*	Disease-Free Survival HR (95% CI)	*p*
Age	1.01 (0.98–1.04)	0.431	1.01 (0.98–1.04)	0.427
Gravida	1.04 (0.89–1.20)	0.645	1.03 (0.89–1.19)	0.684
Parity	1.08 (0.90–1.29)	0.399	1.10 (0.92–1.30)	0.291
Menopausal status	0.68 (0.37–1.24)	0.209	0.75 (0.41–1.36)	0.339
Comorbidity	1.49 (0.85–2.60)	0.162	1.30 (0.75–2.24)	0.352
HPV status	0.98 (0.69–1.37)	0.888	1.10 (0.79–1.54)	0.579
FIGO stage	1.17 (1.00–1.36)	0.051	1.10 (0.94–1.29)	0.225
Adjuvant chemotherapy	0.87 (0.48–1.57)	0.640	1.05 (0.58–1.90)	0.868
Adjuvant radiotherapy	0.92 (0.28–3.00)	0.896	1.04 (0.32–3.37)	0.954
Lymphovascular invasion	1.77 (0.44–2.34)	0.352	1.71 (0.41–2.24)	0.229
Surgical approach	1.86 (0.81–4.26)	0.143	2.28 (0.94–5.56)	0.069
Tumor size	1.13 (0.82–1.55)	0.454	1.12 (0.81–1.55)	0.479
Histological subtype	1.03 (0.51–2.08)	0.938	1.12 (0.55–2.28)	0.763
TROP2 expression	2.01 (1.12–3.58)	0.019	2.97 (1.56–5.64)	<0.001
HER3 expression	1.69 (0.96–2.98)	0.071	1.45 (0.83–2.54)	0.197

**Table 4:** Multivariable Cox proportional hazards regression analyses for overall survival.

Variable	Model 1 HR (95% CI)	*p*	Model 2 HR (95% CI)	*p*	Model 3 HR (95% CI)	*p*
Age	1.07 (1.02–1.13)	0.011	1.07 (1.01–1.13)	0.013	1.07 (1.01–1.13)	0.015
Menopausal status	0.46 (0.09–2.39)	0.358	0.53 (0.11–2.70)	0.448	0.54 (0.11–2.74)	0.455
FIGO stage	1.80 (1.33–2.43)	<0.001	1.70 (1.25–2.30)	<0.001	1.70 (1.24–2.33)	<0.001
Histological subtype	0.31 (0.03–2.88)	0.303	0.38 (0.04–3.64)	0.400	0.38 (0.04–3.77)	0.411
Tumor size	1.22 (0.68–2.21)	0.502	1.34 (0.73–2.48)	0.344	1.35 (0.70–2.60)	0.365
Lymphovascular invasion	1.06 (0.01–1.31)	<0.001	1.06 (0.01–1.32)	0.001	1.06 (0.01–1.32)	0.001
Adjuvant chemotherapy	2.00 (0.61–6.52)	0.252	2.23 (0.66–7.56)	0.199	2.20 (0.62–7.84)	0.223
TROP2	—	—	2.40 (0.61–9.47)	0.213	2.42 (0.59–10.00)	0.221
HER3	—	—	—	—	0.96 (0.27–3.39)	0.950

**Table 5:** Multivariable Cox proportional hazards regression analyses for disease-free survival.

Variable	Model 1 HR (95% CI)	*p*	Model 2 HR (95% CI)	*p*	Model 3 HR (95% CI)	*p*
Age	1.06 (1.01–1.11)	0.026	1.06 (1.01–1.11)	0.025	1.05 (1.01–1.11)	0.028
Menopausal status	0.65 (0.16–2.62)	0.549	0.82 (0.21–3.17)	0.777	0.84 (0.22–3.25)	0.802
FIGO stage	1.56 (1.18–2.06)	0.002	1.42 (1.08–1.87)	0.012	1.45 (1.08–1.93)	0.012
Histological subtype	0.64 (0.12–3.35)	0.602	0.94 (0.17–5.02)	0.939	0.99 (0.18–5.38)	0.988
Tumor size	1.23 (0.74–2.06)	0.428	1.37 (0.81–2.31)	0.237	1.45 (0.80–2.65)	0.224
Lymphovascular invasion	1.09 (0.02–1.35)	<0.001	1.08 (0.02–1.36)	0.001	1.08 (0.02–1.36)	0.001
Adjuvant chemotherapy	2.03 (0.69–5.96)	0.197	2.50 (0.81–7.73)	0.112	2.40 (0.76–7.61)	0.136
TROP2	—	—	4.66 (1.26–17.17)	0.021	5.00 (1.28–19.57)	0.021
HER3	—	—	—	—	0.80 (0.25–2.52)	0.701

## Data Availability

The datasets used in this study can be made available by the corresponding author upon reasonable request, with permission from the Obstetrics and Gynecology Department of Sivas Cumhuriyet University School of Medicine.

## Abbreviations

ADC	antibody–drug conjugate
CI	confidence interval
DFS	disease-free survival
FFPE	formalin-fixed paraffin-embedded
FIGO	International Federation of Gynecology and Obstetrics
HER3	human epidermal growth factor receptor 3
HR	hazard ratio
IHC	immunohistochemistry
IRS	immunoreactivity score
LVSI	lymphovascular space invasion
OS	overall survival
PI3K	phosphatidylinositol 3-kinase
AKT	protein kinase B
MAPK	mitogen-activated protein kinase
ERK	extracellular signal-regulated kinase
TROP2	trophoblast cell surface antigen 2
